# In Silico Approaches for the Identification of Aptamer Binding Interactions to *Leptospira* spp. Cell Surface Proteins

**DOI:** 10.3390/tropicalmed8020125

**Published:** 2023-02-18

**Authors:** Chembie A. Almazar, Marjo V. Mendoza, Windell L. Rivera

**Affiliations:** Pathogen-Host-Environment Interactions Research Laboratory, Institute of Biology, College of Science, University of the Philippines Diliman, Quezon City 1101, Philippines

**Keywords:** *Leptospira*, in silico, aptamer, molecular docking, binding interactions

## Abstract

Aptamers are nucleic acids that can bind with high affinity and specificity to a range of target molecules. However, their functionality relies on their secondary and tertiary structures such that the combination of nucleotides determines their three-dimensional conformation. In this study, the binding mechanisms of candidate aptamers and their interactions with selected target proteins found in the cell surface of *Leptospira* were predicted to select high-affinity aptamers. Four aptamers were evaluated through molecular modeling and docking using available software and web-based tools, following the workflow previously designed for in silico evaluation of DNA aptamers. The most predominant and highly conserved surface-exposed proteins among pathogenic *Leptospira* species were used as aptamer targets. The highest number of interactions was seen in aptamers AP5 and AP1. Hydrogen bonds, along with a few hydrophobic interactions, occur in most aptamer–protein complexes. Further analysis revealed serine, threonine, glutamine, and lysine as main protein residues. H-bond interactions occur mostly with polar amino acids, as reflected in the predicted interaction profiles of aptamer–protein complexes. In silico strategies allowed the identification of key residues crucial in aptamer–target interaction during aptamer screening. Such information can be used in aptamer modification for improved binding affinity and accuracy for diagnostics application.

## 1. Introduction

Increasing attention on aptamer research paved the way to the development of in silico approaches for the selection and design of aptamers through advanced computational methods. Aptamers are short, single-stranded oligonucleotides (DNA or RNA) that form three-dimensional (3D) conformations that allow them to bind to a multitude of targets, from small molecules to complex structures. Given their unique characteristics, specifically, that they possess a wide array of targets, high specificity and affinity, ease of synthesis and modification, high reproducibility with low batch-to-batch variation, and better stability, their application on diagnostics, therapeutics, and other research fields is now being extensively explored [1].

Despite the growing interest in aptamer research, there remains a paucity of high-affinity aptamers for clinical transformation. Aptamers are generally screened through systematic evolution of ligands by exponential enrichment (SELEX), an iterative process that involves evolution, purification, and enrichment of nucleic acids from a random library pool that binds to the target molecule with high specificity and affinity [2]. However, SELEX mechanisms are typically long and exhaustive, requiring optimization to maximize aptamer binding affinity. Because of these challenges, both analytical and in silico approaches were introduced to improve the aptamer selection process. Most notable analytical methods developed applied combinatorial chemistry of SELEX along with capillary electrophoresis [3], gel-based diffusion [4], or microarray [5]. The utility of these platforms together with in silico strategies has circumvented blind aptamer selection and shortened the screening time [1].

In silico strategies using various computational and simulation tools significantly contributed to the advancement of aptamer screening and validation. Early works on aptamer design focused on minimizing the number of sequences of the starting library, which normally uses approximately 10^15^ random nucleic acid sequences. The preselection procedure using bioinformatics tools limited the sequences to those with a high potential for binding to the target, thus ensuring that candidate aptamers enriched during each selection round have higher binding affinity and selectivity to the target of interest [6,7]. In addition, molecular docking and modeling have been used to assist in aptamer design and optimization. With the numerous bioinformatics resources available, it is possible to predict the putative secondary and tertiary structures of aptamers and their targets, thereby revealing their thermodynamic properties. Furthermore, simulations of aptamer–target complexes facilitated virtual aptamer screening, identification of structural motifs and key interaction residues, and elucidation of noncovalent interactions necessary in understanding aptamer–ligand affinity [8]. Insights from structural modeling also allowed tailored chemical modification of aptamer sequences to improve the accuracy of their molecular recognition ability and stability [9,10].

Owing to the successful works in aptamer selection through in silico approaches, this study aimed to predict the binding mechanisms of candidate aptamers and their interactions with selected target proteins on the *Leptospira* cell surface. Leptospirosis is considered a re-emerging public health concern worldwide. In fact, in the past decades, increasing incidence has been reported, particularly in developing countries, where numerous outbreaks have occurred, mostly in urban slum areas or after heavy rainfall and flooding [11]. Despite its threat to public health, early diagnosis remains a challenge, which is mainly attributed to the unavailability of testing facilities or lack of rapid point-of-care diagnostic tests. Leptospirosis diagnosis often relies on clinical presentations, sometimes resulting in misdiagnosis because of its resemblance to other endemic diseases [12]. Development of specific and sensitive diagnostic tests is therefore imperative for appropriate patient management and treatment. Using aptamers selected against *Leptospira* from in vitro experiments, we derived their secondary and tertiary structures by adopting a previously described workflow for structural modeling [13]. Aptamer interactions with selected proteins were also predicted to determine possible binding residues. Such information is useful in the optimization of aptamer design for diagnostics application. This study, therefore, intended to explore the primary surface binding factors of the aptamers necessary for the detection of the pathogen during the early stages of the disease, since surface-exposed proteins are key epitopes that serve as aptamer recognition sites for binding.

## 2. Materials and Methods

### 2.1. Aptamers and Leptospira spp. Outer Membrane Protein Targets

Four aptamers generated previously by a third-party service laboratory, Novaptech (France), using the cell-SELEX method against the *Leptospira* vaccine, were used to determine the possible binding sites to the target proteins. Initially, 15 candidate aptamers were selected based on their frequency during the enrichment process. The binding properties of these candidate aptamers were further evaluated by surface plasmon resonance (SPR). Results indicated that the four aptamers had the highest binding signal against the antigen.

Protein targets were selected through a systematic review of available proteomic data from different online databases. The most common and abundant *Leptospira* outer membrane proteins implicated in its pathogenicity and virulence and have been previously selected for vaccine and diagnostic research were used in the study. The amino acid sequence of the selected proteins was then obtained from UniProt. The target proteins used in the study were sourced from various studies on *Leptospira* surface proteins, elucidating their roles in the pathogenicity of the microorganism.

Since most of these proteins are not in the PDB database, amino acid sequences were submitted to the SWISS-MODEL server (https://swissmodel.expasy.org/, accessed on 30 June 2022) to generate the in silico 3D structures, except for the LipL32 protein [14]. The model template for each protein was then used as the receptor molecule during docking.

### 2.2. Software Workflow

**Figure 1:**
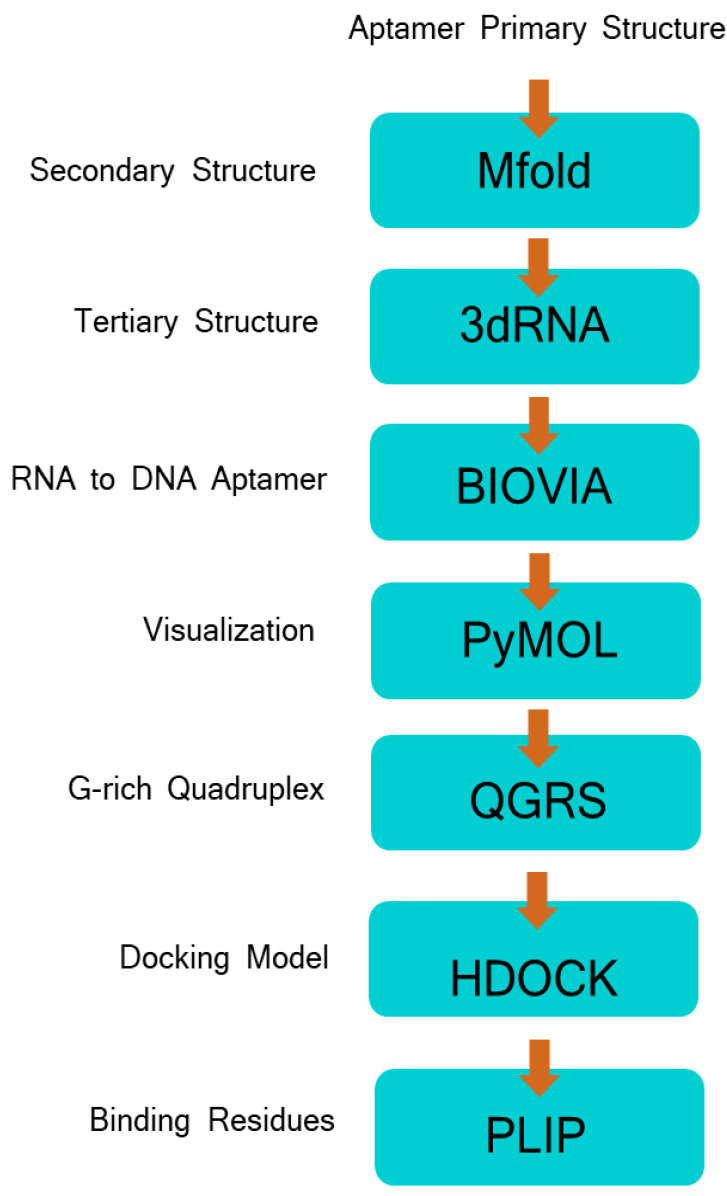
Bioinformatics pipeline to predict the 3D structure of aptamers and the docking model, including the identification of significant interactions on aptamer–protein target complexes.

A workflow based on free bioinformatics tools validated by Oliviera et al. [13] in 2022 was utilized to predict the binding residues associated with the interaction of aptamers to target proteins of the *Leptospira* spp. (Figure 1). Using DNA aptamers as the starting point, the nucleotide sequence of aptamers was used to predict the secondary structure using the Mfold web server (http://www.unafold.org/mfold/applications/dna-folding-form.php, accessed on 4 July 2022) and default values and parameters [15]. Ionic conditions were set as [Na+] = 1.0 M and [Mg2+] = 0.0 M. For each aptamer, the most thermodynamically stable structure (lowest free Gibbs energy) was selected, and the corresponding Vienna file (dot bracket format [.b file]) was saved.

The tertiary structure of each aptamer was assembled using 3dRNA using the Vienna file input from the previous analysis [16]. However, the 3dRNA web server (http://biophy.hust.edu.cn/new/3dRNA, accessed on 4 July 2022) was developed for RNA structures; hence, the thymine (T) from the sequence was replaced by uracil (U). All analyses were performed using the Procedure Optimize, 5 predictions, 3dRNA-Lib1, and minimization parameters, and results were saved as PDB files.

Conversion of the RNA structures back to DNA was performed using Biovia Discovery Studio Visualizer software [17]. Conversion was performed by substituting the uracil (U) nucleotide to thymine (T) and by changing the pentose sugar from ribose to deoxyribose.

All structures were then imported to PyMOL to add hydrogen atoms, which play an important role in molecular docking and interaction. Prediction of the G-rich quadruplexes were carried out using the QGRS mapper (https://bioinformatics.ramapo.edu/QGRS/index.php, accessed on 3 August 2022). This software maps the location of potential G-quadruplexes, instrumental in the stability of the 3D structure of the aptamer, in each nucleotide sequence [18].

Finally, the 3D structures of the aptamers were saved as PDB files and used as the input for receptor–ligand docking. This process was also repeated for the protein targets prior to docking. The docking simulation was performed on the HDOCK web server (http://hdock.phys.hust.edu.cn/data/62bd3e9f971c2/, accessed on 6 July 2022), using the tertiary structure of the aptamer as the ligand input and the PDB file of the protein target as the receptor input [19].

The best docking model (lowest docking energy score) was selected and used for the identification of binding sites with the PLIP web server (https://projects.biotec.tu-dresden.de/plip-web/plip, accessed on 16 August 2022) [20]. Noncovalent interactions, such as hydrogen bonds and hydrophobic interactions, were recorded and interacting amino acids were identified.

### 2.3. Validation of Aptamer-Target Binding

Direct ELAA was performed using *Leptospira* vaccine as antigen (Vanguard Plus 5, Zoetis CA). Vaccine components include a separate vial containing *Leptospira canicola* and *Leptospira icterohemorraghiae* bacterin, in which concentration was determined to be 2.93 × 10^9^ cells/mL. The inactivated cells were concentrated via centrifugation at 10,000× *g* for 1 min at 4 °C. Flat-bottomed 96-well plates were coated with 100 µL of the 2.00 µg/µL antigen in 0.1 M carbonate buffer incubated overnight at 4 °C. Blocking was performed using 5% *w/v* skimmed milk powder in PBS-Tween (Sigma-Aldrich, MI, USA) buffer for an hour at room temperature. Fifty microliters of 50 pmol biotinylated aptamer were subsequently added. Direct detection was performed by adding 50 µL of 1:10,000 *v/v* freshly prepared streptavidin-peroxidase; 50 µL TMB (Sigma-Aldrich, MI, USA) was used as the substrate. Reaction was terminated using 50 µL concentrated H_2_SO_4_ (36N). All steps required a 30 min incubation immediately followed by washing using PBS-Tween. Absorbance was recorded at 450 nm. One-way ANOVA test using GraphPad Prism 8 was performed to determine the significance of the capability of aptamers to detect the antigen against the negative control with Tukey’s test for multiple comparisons as the post hoc test.

## 3. Results

### 3.1. Aptamer–Target Proteins and Their Predicted Structures

Proteins are popular targets of aptamer design and modeling. In the present study, through an extensive search of the literature, *Leptospira* outer membrane proteins implicated in their pathogenicity were chosen as potential aptamer targets. Table 1 shows 11 proteins, commonly known as cell surface proteins, found in pathogenic strains that cause human infection and have been mostly used in vaccine or diagnostic development research. Among the selected proteins is LipL32, which was found to be a good diagnostic indicator of leptospirosis [21]. However, the structural information of the target proteins has been limited to LipL32 alone. The 3D structures of the other proteins were generated using SWISS-MODEL through homology modeling. Results revealed less than ideal structures with low model scores, which can be due to the lack of available protein structures in the Protein Data Bank (PDB) website that serves as the template for protein modeling, prediction, and analysis.

### 3.2. Secondary and Tertiary Structures of Candidate Aptamers

**Figure 2:**
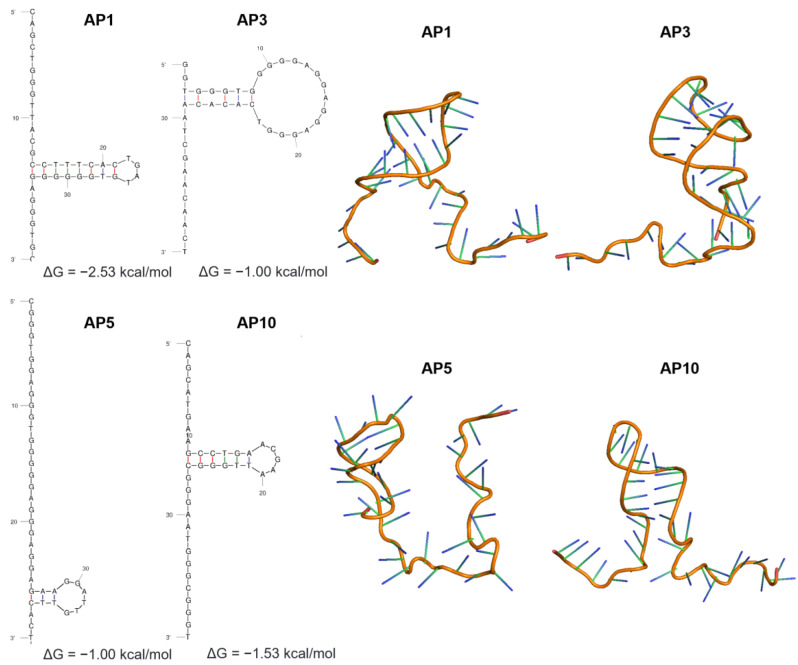
Secondary and tertiary structures of 40 mer aptamer sequences (AP1, AP3, AP5, and AP10) screened via cell-SELEX against inactivated *Leptospira* cells. The most thermodynamically stable structures (lowest free Gibbs energy) predicted using the MFold program were selected.

Because no experimentally resolved structures were found in the PDB database for the DNA aptamers against *Leptospira* spp., four aptamers previously generated through cell-SELEX were used in this study. The ssDNA nucleotide sequences of the aptamers were used as the starting point to build their secondary and tertiary structures. Figure 2 shows the identical secondary structures of the aptamers, all consisting of single-stranded segments at the 5′ and 3′ ends and one small hairpin stem-loop structure in between. Mfold analysis calculated the Gibbs free energy, ΔG, ranging from −0.10 to −2.53 kcal/mol, with AP1 having the lowest energy. Putative quadruplexes forming G-rich sequences (QGRS) were also predicted using the QGRS mapper (22). The calculated G-scores were 20 (AP1), 21 (AP3), and 40 (AP5 and AP10). The high G-scores of AP5 and AP10 suggest that these aptamers have the most stable G-quadruplex motif.

Accordingly, the predicted tertiary structures using 3dRNA displayed shared patterns save for the orientation of the hairpin loop in the structures. This folding pattern is attributed not only to the nucleotide sequence composition but also to the conditions (ionic environment and temperature) from which these were determined. Tertiary structures with the lowest score were used in the modeling of aptamer–protein complexes.

### 3.3. Evaluation of the Binding Capacity of Aptamers by Direct Enzyme-Linked Aptamer Assay (ELAA)

**Figure 3:**
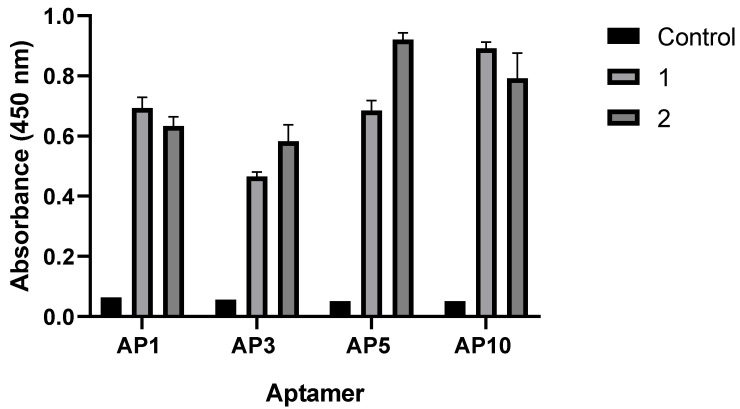
Detection of *Leptospira* antigen using four candidate aptamers via direct ELAA. Absorbance reading was at 450 nm. One-way ANOVA indicates a significant difference between control and test groups at *p* < 0.0001. Tukey’s post hoc multiple comparisons test: control vs. 1 (*p* = 0.0002) and control vs. 2 (*p* = 0.0001).

Direct ELAA was performed to test the binding affinity of four candidate aptamers against a commercially available *Leptospira* vaccine. Results show strong signals from all candidate aptamers, indicating the possible affinity of the aptamers to the target antigen, with AP5 and AP10 having the highest signal (Figure 3). A one-way ANOVA test was performed to compare the absorbance results of the assay relative to the negative control. The test revealed that there is a significant difference in mean absorbance values between the test and control groups (F(2,9) = 31.69, *p* < 0.0001). Tukey’s multiple comparisons test indicated that the mean value of absorbance was significantly different between the control group and test group 1 (*p* = 0.0002, 95% C.I.= −0.8824 to −0.3734) and test group 2 (*p* = 0.0001, 95% C.I.= −0.9310 to −0.4220). Results of this analysis indicate that direct ELAA for these aptamers has significant absorbance values from the negative control that warrants further studies for diagnostic research and development.

Evaluation of the binding interactions to specific cell surface proteins, however, needs to be carried out to validate which proteins form a strong interaction to the aptamers. Furthermore, experiments involving the specificity of the chosen aptamers to *Leptospira* may need to be performed to eliminate cross-reactivity to other blood-borne pathogens. Since this is only an exploratory study that deals with simulating all the possible interactions that may occur between the aptamer and the *Leptospira* target proteins, the limitations of this study were acknowledged and will be addressed in future studies. 

### 3.4. Aptamer and Leptospira Cell Surface Protein Complexes and Their Interacting Residues

**Figure 4:**
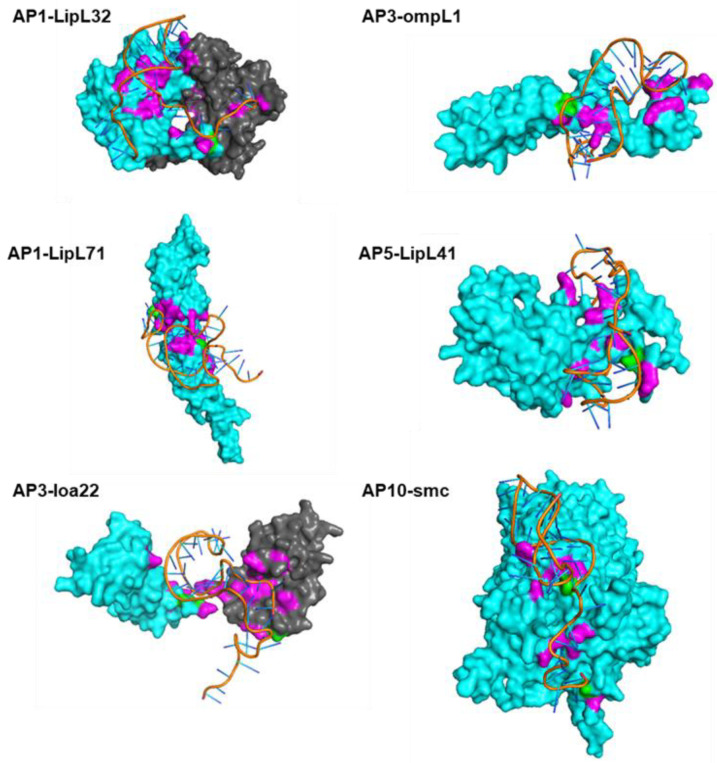
Predicted binding sites of aptamers with the highest number of interactions to six selected target proteins, using HDOCK and visualized through PyMOL. Simulated aptamer–protein complexes show hydrogen bonds in pink and hydrophobic interactions in green.

Molecular docking simulations were performed via HDOCK. Using the tertiary structure of the aptamer and the template model of the protein as inputs, interactions between the receptor and ligands were then investigated. Although several docking models were generated, the best models for each aptamer–protein pair were selected using a scoring function that indicates the quality of the docking model. The model with the lowest docking score was selected for the identification of the mechanisms of binding between the aptamer and the protein target. Figure 4 shows some of the representative models of the molecular docking carried out using HDOCK, with hydrogen bonds highlighted in pink and hydrophobic interactions highlighted in green.

**Figure 5:**
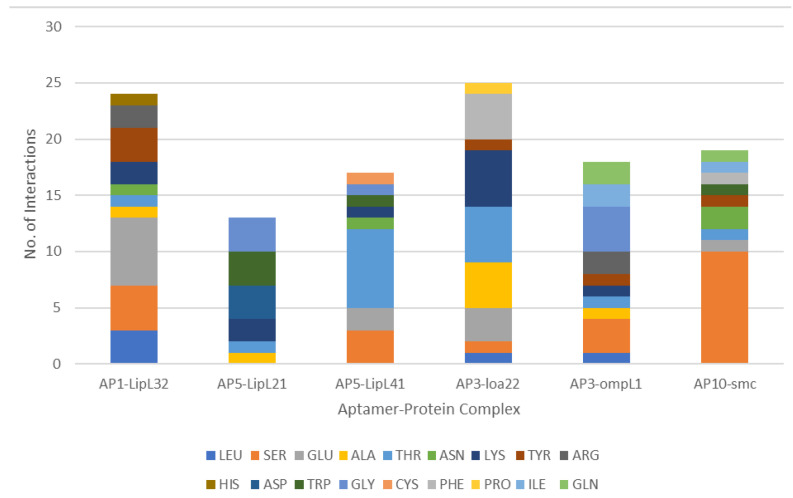
Number of interactions on the selected aptamer–protein model complexes.

Noncovalent interactions between the aptamer ligand and protein receptor were identified using the Protein–Ligand Interaction Profiler (PLIP) web server (Table S1). Overall, AP5 had the highest number of interactions, with 20 hydrophobic interactions and 152 hydrogen bonds identified; meanwhile, the AP1, AP3, and AP10 aptamer–protein complexes had a total of 170, 159, and 155 predicted interactions, respectively. Given these profiles, the aptamer–protein complex with the highest number of interactions for the six selected proteins (LipL32, LipL71, LipL41, OmpL1, Loa22, and Smc) was further evaluated. The number of protein interactions with specific amino acids that bind to the aptamers was identified (Figure 5). Analysis of the interacting residues showed serine as the most abundant protein residue, at 18.10% of all the total interactions in all the model complexes, followed by threonine (13.79%), glutamine (10.34%), and lysine (9.48%). However, arginine and histidine, which are positively charged amino acid residues, comprised 3.45% and 0.86% of the total interactions, a finding that challenges the idea of binding interactions between negatively charged aptamers and positively charged amino acid residues. The interactions identified in this study can be helpful in elucidating the mechanisms of binding of the aptamer to its target protein as well as contributing to the aptamer–protein complex stabilization.

## 4. Discussion

A previously designed bioinformatics pipeline that used free available web-based servers and software was adopted to predict DNA aptamers’ secondary and tertiary structures and their complexes with target proteins [13]. Utilization of this workflow allowed simulation of interactions of candidate aptamers with *Leptospira* cell surface proteins necessary in devising an aptamer-based diagnostic test kit. Recent studies have shown the significant contribution of computational tools in the development and optimization of aptamers before and after SELEX. The in silico approach uses various computational tools for structure prediction, molecular docking of aptamers to target molecules, and statistical analysis to evaluate the binding affinity and selectivity of aptamers [33]. Implementation of such a strategy along with experimental procedures improves the cost-efficiency rate of aptamer screening and design.

Proteins located in the outer membrane of the cell are potential targets for aptamer binding. Among pathogenic species of *Leptospira* are surface-exposed outer membrane proteins that confer virulence to the microorganism. These surface-exposed antigens are likely involved in the primary host–pathogen interactions that result in tissue adhesion, immune response cascades, invasion, and eventual evasion of the host immune system [30]. The LipL protein family, specifically, has been implicated in the bacterial virulence and host interactions. Among these LipL proteins are LipL21, LipL32, LipL41, and LipL71, which are reported as the most abundant proteins in the leptospiral surfaceome [34,35]. LipL32 has been implicated in the stimulation of inflammatory responses, as evident in its high expression levels during infection [36]. LipL21 and LipL41 play key roles in pathogenic interactions with several host components contributing to successful *Leptospira* colonization [37]. LipL71, on the other hand, is a surface-exposed lipoprotein that modulates cellular interactions during leptospiral pathogenesis [38]. These proteins, along with the others listed in Table 1, were tagged as potential targets for reverse vaccinology and diagnostic tests for leptospirosis [32,39,40]. However, surface-exposed epitopes are not predicted owing to the limited data on the protein targets.

Aptamers are highly flexible molecules that can readily change their configuration depending on the conditions under which they are introduced [21]. The secondary structure of the aptamers is attributed to intramolecular nucleotide base pairing, which allows the molecule to fold in certain conformations [33]. Mfold predictions largely rely on the minimum free energy (ΔG) as Mfold’s core algorithm along with the minimum free energies for the interaction of base pairs where the conformation with lowest ΔG value was selected [15]. Factors affecting the folding of the aptamers, including temperature and ionic concentrations, were also considered; however, both are fixed parameters in Mfold and, thus, cannot represent the environmental conditions in vivo [15]. Mimicking the highly complex SELEX conditions is still a challenge in in silico molecular docking of aptamers and target proteins.

The binding affinity and specificity of aptamers to their targets are highly dependent on their 3D structure. Aptamers are folded into unique structures possessing a combination of hairpins, loops, pseudoknots, and G-quadruplexes that anchors aptamers to surface epitopes of the target proteins [41,42]. These structures can be attributed to the flexibility of the phosphodiester backbone of aptamers that confers different torsional angles and enables the generation of a wide variety of tertiary structures [43]. Hence, aptamers can exist in different conformations in solution along with varied binding affinities to the target protein. However, computational tools for the prediction of the 3D structure of aptamers are still limited to RNA-based software applications. The introduced integrated pipeline allows for the prediction of the tertiary structure of DNA aptamers using their RNA equivalents. A study by Jeddi and Saiz [44] confirmed that structural conversion between DNA and RNA molecules in silico produces identical aptamer hairpin conformations. Thus, by simply replacing thymine with uracil in the nucleotide sequence, the 3D structure of the aptamers can be visualized through the 3dRNA software. The RNA tertiary structures, thus, can be reverted into DNA structures by the modification of the sugar residues (2′ –OH to 2′ –H), the bases (uracil to thymine), and the sugar backbone (ribose to deoxyribose).

Having determined the tertiary structures of the aptamers, the structures were subjected to molecular docking. This is a computational method to predict the formation of the aptamer–protein complex based on the lowest ΔG docking scores [6]. Docking algorithms can be divided into two categories, namely, template-based algorithm and machine learning algorithm [9]. In the case of the HDOCK server, both algorithms are considered when molecular docking is being performed through a hybrid strategy of template-based modeling and ab initio template-free docking [19]. Since most of the structures used in this study are absent in databases, the server can opt to perform the docking based on available sequence and structural information input alone. One of the limitations of this study is that, since these structures are not experimentally resolved, the interactions are only the approximation of all possible binding interactions between the aptamer and the target protein. Nevertheless, the binding sites for these aptamer–protein interactions can now be determined through an array of structural motifs. Based on these conformations, aptamers can bind to protein targets via hydrogen bonding, hydrophobic interactions, electrostatic interactions, van der Waals forces, and π–π stacking [45].

Surface protein epitopes recognized by aptamers are mostly electropositive and dominated by polar interactions, hydrogen bonds, and charge–charge interactions, aside from the interactions involved in base stacking [46]. However, hydrophobic interactions, known to make key contributions to protein–protein interactions, are limited in native nucleic acid ligands. This limitation has been circumvented by the addition of pre- and post-SELEX modifications to the nucleotide structure that enables high-affinity binding to the target protein [42]. However, in this study, the native 3D structure of the aptamers was utilized; hence, predominantly, hydrogen bonds and a few hydrophobic interactions to the amino acid side chains were observed. Nevertheless, these interactions are instrumental in determining the binding affinity of the aptamer to the target protein. Hydrogen bonding is the most common surface interaction between aptamers and target molecules where the amino acid side chains of the target molecule act as H-bond donors and the negatively charged phosphate backbones act as H-bond acceptors [47]. This interaction contributes much to the binding force stabilizing the aptamer–protein complex, in which most of the H-bond interactions occur with polar amino acids such as serine, threonine, and glutamine residues, to name a few [43,48]. By contrast, hydrophobic interactions occur on nonpolar amino acid residues and are often observed in protein–protein interactions, including aptamers modified with novel functional groups engineered to increase their binding affinity to target molecules [49].

The implementation of the algorithm for the aptamer–protein complex identified the best docking model using the predicted tertiary structures of the DNA aptamers and protein models. AP1 and AP5 had the highest number of interactions; however, the relative abundance of the proteins evaluated was not taken into consideration (Table S1). As mentioned, LipL32, LipL41, LipL21, OmpL1, Smc, and Loa22 are among the most predominant surface-exposed proteins that are highly conserved among pathogenic *Leptospira* spp. Specifically, LipL32 and LipL41 are reported as the most abundant proteins with high diagnostic accuracy [32,34,50]. As there is no information on the differential abundance of each evaluated proteins, the authors worked on the assumption that LipL32 and LipL41 are the most abundant surface proteins among these proteins. All aptamers were noted to have the high number of interactions with these two proteins, specifically, AP1 had the highest affinity, followed by AP10, AP5, and AP3, with a total of 38, 34, 33, and 20 interactions, respectively. Given this information, AP1, AP10 and AP5 can be considered for the development of an aptamer-based diagnostic kit, since these aptamers had the highest number of interactions with LipL32 and LipL41, as supported by the direct ELAA results. Further validation studies are recommended to determine the best performing aptamer. 

Results of the assay indicated that these aptamers were able to sufficiently detect the antigen which is indicative of their performance for diagnostic use. However, further experiments need to be performed to validate these findings. In addition to sensitivity, specificity is one of the most important parameters that defines the performance of the analytic assay [42]. Experiments that evaluate the specificity of these aptamers to *Leptospira* are essential to limit its cross-reactivity to other blood-borne pathogens and competition with other host ligands. As most *Leptospira* proteins remain unknown, the application of the cell-SELEX method offers an advantage, such that selected aptamers are expected to bind to the cell surface proteins in their native conformations, which are often difficult to chemically synthesize [6].The ability of these cell-specific aptamers to bind to a multiple target may be considered an essential feature, as they could increase the sensitivity of the assay. Binding affinities of aptamers to target proteins should also be taken into consideration to improve its diagnostic capability. 

Preliminary data obtained from this study revealed key binding residues that can be explored further to improve the affinity of the aptamer to the target and, at the same time, identify nucleotides that can be modified to improve binding conformation of the aptamers, as performed in a previous study [10]. Aptamer mutation can also be achieved in silico in order to maximize its binding capacity to target ligands and to eliminate the possibility of binding to host proteins. Interactions between aptamers and their ligands are complex and difficult to evaluate using in vitro experiments alone; thus, complementary in silico aptamer design is important to facilitate screening of aptamers as well as increase our understanding of nucleic acid–protein interactions [42]. Combinatorial in silico aptamer design in conjunction with in vitro SELEX experiments proved to be effective in designing aptamers for thrombin detection [51] as well as for SARS-CoV-2 spike protein [52], enabling the development of these potent tools for diagnostics. Furthermore, this workflow permits the simulation of aptamers binding to different matrices and other structurally related compounds; thus, efficient modification may be employed to limit its cross-reactivity. Despite the limitations of this study, employing a bioinformatics approach is still central to the development of aptamer-based detection technologies.

## 5. Conclusions

A bioinformatics pipeline was utilized to determine the interactions between aptamers and the surface proteins of *Leptospira* spp. Three aptamers, AP1, AP5 and AP10, can be considered for the development of aptamer-based diagnostic tests, based on their interactions to the surface proteins highly involved in the pathogenicity of the organism. These aptamer–protein complexes, simulated through molecular docking, were able to demonstrate the anchoring of the aptamers to the protein surface as well as determine the key residues necessary for their stability. However, further studies should be considered to evaluate the binding affinities of these aptamers to the target proteins including, but not limited to, the addition of pre- or post-SELEX modifications to enhance binding capacities. Despite the limitations, the in silico approach is still a powerful method to expand our knowledge on DNA aptamer–protein interaction and presents promising strategies for the development of aptamer technology.

## Figures and Tables

**Table 1:** Selected *Leptospira* outer membrane proteins used in this study.

Gene/Protein Target	Function	UniProt ID	PDB File Available?	Used for Vaccine or Diagnostics
HlyX	Hemolysis [22]	Q72VH2	No	No
LIC12575	Outer membrane efflux protein [23,24]	Q72PA0	No	Yes
LIC20151	TonB-dependent outer membrane receptor [23,24]	Q75FN1	No	Yes
LigA	Immune response [25]	G1UB65	No	Yes
LipL21	Virulence [26]	Q72WC6	No	Yes
LipL32	Inflammatory response [27]	Q72SM7	Yes	Yes
LipL41	Virulence [28,29]	Q72N71	No	Yes
LipL71	Virulence [29]	Q72TL5	No	Yes
*loa22*	Virulence [30]	Q7X5A5	No	Yes
*ompL1*	Outer membrane protein [29,31]	Q72TP4	No	Yes
*smc*	Outer membrane receptor [32]	Q72UE3	No	No

## Data Availability

Protein sequences are available in the Uniprot database (https://www.uniprot.org/ accessed on 15 February 2023). Aptamer sequences are shown in Figure 2. Further inquiries can be directed to the corresponding author.

## Supplementary Materials

The following supporting information can be downloaded at: https://www.mdpi.com/article/10.3390/tropicalmed8020125/s1, Table S1: Aptamer–protein interaction profiles.
