# Supplementary material for: In Silico Approaches for the Identification of Aptamer Binding Interactions to Leptospira spp. Cell Surface Proteins
Source: Trop Med Infect Dis. 2023 Feb 18;8(2):125. doi: 10.3390/tropicalmed8020125 (PMC9963831; doi:10.3390/tropicalmed8020125)
Supplement: Supplementary file 1 [file tropicalmed-08-00125-s001.zip › tropicalmed-2163468-supplementary.pdf]

Supplemetary Material: In Silico Approaches for the Identification of Aptamer Binding Interactions to *Leptospira* spp. Cell Surface Proteins

Chembie A. Almazar †, Marjo V. Mendoza † and Windell L. Rivera *

Pathogen-Host-Environment Interactions Research Laboratory, Institute of Biology, College of Science, University of the Philippines Diliman, Quezon City 1101, Philippines; caalmazar@up.edu.ph (C.A.A.); mvmendoza3@up.edu.ph (M.V.M.)
* Correspondence: wlrivera@science.upd.edu.ph
† These authors contributed equally to this work.

Table S1. Aptamer–protein interaction profiles.

Aptamer	Protein	No. of hydrophobic interactions	No. of hydrogen bonds	Total no. of interactions
AP1	HylX	2	13	15
AP1	LIC12575	0	12	12
AP1	LIC20151	4	10	14
AP1	LigA	0	18	18
AP1	LipL21	0	12	12
AP1	LipL32	1	23	24*
AP1	LipL41	1	13	14
AP1	LipL71	2	20	22*
AP1	Loa22	3	10	13
AP1	OmpL1	1	10	11
AP1	Smc	3	12	15
Total		17	153	170
AP3	HylX	3	10	13
AP3	LIC12575	1	9	10
AP3	LIC20151	1	17	18
AP3	LigA	3	16	19

AP3	LipL21	0	6	6
AP3	LipL32	0	8	8
AP3	LipL41	3	9	12
AP3	LipL71	1	12	13
AP3	Loa22	3	22	25*
AP3	OmpL1	1	17	18*
AP3	Smc	1	16	17
Total		17	142	159
AP5	HylX	3	13	16
AP5	LIC12575	1	16	17
AP5	LIC20151	3	19	22
AP5	LigA	2	12	14
AP5	LipL21	2	11	13
AP5	LipL32	0	16	16
AP5	LipL41	1	16	17*
AP5	LipL71	0	12	12
AP5	Loa22	3	14	17
AP5	OmpL1	3	6	9
AP5	Smc	2	17	19
Total		20	152	172
AP10	HylX	2	12	14
AP10	LIC12575	0	13	13
AP10	LIC20151	3	1	4
AP10	LigA	1	15	16
AP10	LipL21	3	8	11
AP10	LipL32	2	17	19

AP10	LipL41	2	13	15
AP10	LipL71	0	19	19
AP10	Loa22	2	9	11
AP10	OmpL1	3	11	14
AP10	Smc	2	17	19*
Total		20	135	155

*Highest number of interacting residues for each protein.
